# Uniaxial Rotational Molding of Bio-Based Low-Density Polyethylene Filled with Black Tea Waste

**DOI:** 10.3390/ma16103641

**Published:** 2023-05-10

**Authors:** Joanna Aniśko, Mateusz Barczewski

**Affiliations:** Institute of Materials Technology, Poznan University of Technology, Piotrowo 3, 61-138 Poznan, Poland

**Keywords:** rotational molding, bio-polyethylene, black tea, waste filler, composites

## Abstract

In this paper, the possibility of obtaining uniaxially rotomolded composite parts was discussed. The used matrix was bio-based low-density polyethylene (bioLDPE) filled with black tea waste (BTW) to prevent the thermooxidation of samples during processing. In rotational molding technology, the material is held at an elevated temperature in a molten state for a relatively long time, which can result in polymer oxidation. The Fourier transform infrared spectroscopy (FTIR) shows that adding 10 wt% of black tea waste has not led to the formation of carbonyl compounds in polyethylene, and adding 5 wt% and above prevents the appearance of the C–O stretching band connected with degradation of LDPE. The rheological analysis proved the stabilizing effect of black tea waste on the polyethylene matrix. The same temperature conditions of rotational molding did not change the chemical composition of black tea but slightly influenced the antioxidant activity of methanolic extracts; the detected changes suggest degradation is a color change, and the total color change parameter (ΔE) is 25. The oxidation level of unstabilized polyethylene measured using the carbonyl index exceeds 1.5 and gradually decreases with the addition of BTW. The BTW filler did not influence the melting properties of bioLDPE; the melting and crystallization temperature remained stable. The addition of BTW deteriorates the composite mechanical performance, including Young modulus and tensile strength, compared to the neat bioLDPE.

## 1. Introduction

Rotational molding technology is characterized by low shear rates during processing and relatively low costs of tools and equipment [1]. This uncomplicated polymer processing technology allows for obtaining thin walls and ready-to-use hollow products [2] without additional post-processing like chemical tanks, kayaks, children’s play furniture, and toys [3]. The typical rotational molding process is operated using a biaxial rotational machine, which rotates mold on a vertical and horizontal axis at low speed to avoid centrifugal forces throwing plastic against the mold walls. The ratio of used rotational speeds is usually 4:1 [3]. The rotational molding process can also be operated on uniaxial laboratory machines, giving even simpler processing technology. Simultaneously, this special type of rotational molding became a powerful tool that allows us to observe how powder materials behave while rotating in a glass mold. Five different behaviors can be distinguished during the first step of processing: slipping, avalanching, rolling, cataracting, and centrifuging [4]. The powder characteristic affects what kind of behavior will be observed while uniaxial rotational molding [3,5]. For example, the slipping is observed mainly for disk-shaped particles, while frictional forces between the mold surface and powder particles are low [6]. Another two desirable polymer powder flows are avalanching and rolling. Polymer particles more susceptible to avalanche are ‘squared egg’ particles, while the rolling flow occurs for more spherical particles [3,5]. Not only does polymer powder shape impact the flow characteristic, but also rotation speed. With the increase of that parameter, the slipping behavior changes into avalanching, and with a further increase in rotation speed, the powder bed starts to cataract [4].

The uniaxial rotational molding is an excellent way to test materials design for rotomolding, and its application may also be treated as an up-scalable technological test [7]. The most popular polymer used in the rotational molding industry is polyethylene, which has ideal properties for this technology. The uniaxial rotational molding for this material can be successfully conducted [8]. Since the preparation of composite materials in rotational molding technology is receiving increased interest [1,9,10], the works discussing possibilities of preparing those composites in uniaxial rotational molding also increase. One of these kinds of works discusses the production of PE composites filled with hollow glass microspheres (HGM) [10], and another paper concerns the same filler but in a different polymeric matrix, mainly poly(caprolactone) (PCL) [11]. In both these studies, fillers were submitted to a surface treatment, which was a two-step process; firstly, HGM particles were hydroxylated and then silanized to increase the adhesion between the polymer matrix and filler. Preparing good-quality rotomolded composites is complex because this technology has low-shearing conditions, resulting in low mixing [12]. The previously distinguished particle flow characteristic in uniaxial rotational molding also impacts that. To improve the mixing and distribution of filler in rotomolded parts, the two-step preparation process can be applied. In our previous study [13], the introduction of expanded vermiculite into a PE matrix in rotational molding technology was discussed. It was confirmed that the preliminary step concerning melt mixing of polymer with filler helped to obtain parts with increased mechanical properties and better filler distribution than the dry blended composites. When it comes to reinforcing polymer with organic fillers via rotational molding, the chemical modification of fillers is used, usually the silanization [12], hydroxylation [8], or addition of coupling agent, for example, maleated polyethylene [9]. The preparation of composites in rotational molding requires additional chemical preprocessing of fillers or preliminary melt mixing with a polymer matrix to obtain good quality parts, but the preliminary melt mixing can result in thermal or thermomechanical degradation of the matrix or filler because of the additional impact of high processing temperatures. The considered in this study matrix is biobased polyethylene, which is, like the petrochemical one, susceptible to thermooxidation [14].

One of the widely used methods to prevent thermooxidation of polymers is the incorporation of additives containing the synthetic hindered phenols, as commercially available compounds like Irganox 1076 or Irganox 1010, or phenolic antioxidant butylated hydroxytoluene (BHT) [15,16]. The confirmed migration of these compounds from polyethylene to water and foods can raise concerns regarding the hazardous impact on human life and the environment [17]. The naturally available antioxidants have grown an interest considering them as good stabilizers for polyolefins [18]. The most common method is introducing pure antioxidant compounds that have a source in fruits and plants [19,20,21]. The second popular method is incorporating extracts from plants [22] or herbs [23], which are rich in antioxidant compounds and outperform artificial antioxidants. The last possibility for improving polyethylene thermooxidative properties is the direct use of fruit and plant wastes. For example, adding coffee grounds and coffee by-product products into the polyethylene matrix increases the oxygen induction time (OIT) [24]. The same good properties have HDPE filled with cocoa husk [25]. The industry by-products rich in antioxidants also contain cellulose, which is susceptible to thermal degradation. The experiment conducted by Iyer et al. for LDPE composites with grape pomace waste, turmeric waste, orange peel, and coffee grounds shows a decrease in initial degradation temperature (T_10%_) with an increase in addition of waste filler but finally, the next steps of degradation were shifted into the higher temperatures [26].

**Figure 1:**
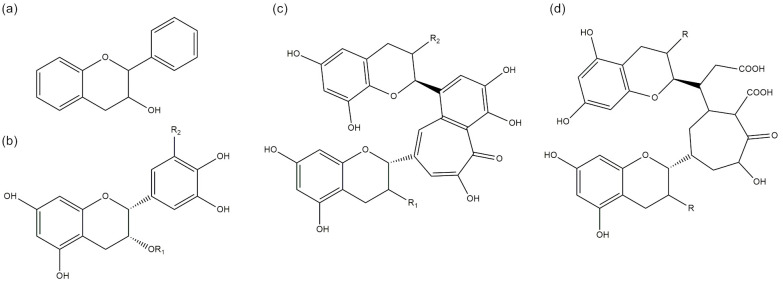
The main compounds derived from black tea: flavanols (**a**), catechins (**b**), theaflavins (**c**), thearubigins (**d**).

In this paper, the addition of industry by-products will be discussed to prevent thermooxidation of polyethylene processed by rotational molding technology. In this case, the used post-production filler was tea dust collected during industrial-scale production. Dried tea leaves contain up to 36% of polyphenolic compounds responsible for all antioxidant properties; tea leaves are the most prominent compound group [27]. The rest of black tea components are carbohydrates, proteins, lignin, ash, amino acids, lipids, organic acids, and chlorophyll. Chemical compounds of interest in this work are polyphenols which can be divided into four main groups: flavanols, catechins, theaflavins, and thearubigins (Figure 1) [28]. The last two groups are characteristic of black tea because of the additional fermentation step require to produce black tea from fresh green tea leaves. The antioxidant activity of catechins extracted from black tea is arranged as follows epigallocatechin-3-gallate (EGCG) > epigallocatechin (EGC) > epicatechin-3-gallate (ECG) > epicatechin (EC) [29]. Theaflavins are dimers of catechins and since they have more -OH groups they can scavenge radicals more effectively than catechins, especially TF3 [27]. Thearubigins in low concentrations showed a higher ability to scavenge hydroxyl radicals than theaflavins and EGCG [30]. Teas rich in theaflavins and thearubigins have good scavenging activity by donating hydrogen atom and teas rich in thearubigins exhibits better ability to scavenge hydroxyl radical [31].

## 2. Materials and Methods

### 2.1. Materials

To prepare composites in a uniaxial rotational molding technology the bio-based low-density polyethylene LDPE SEB 853 I’m Green^®^ obtained from Braskem (São Paulo, Brazil) was used and the waste filler was the black tea wastes coming from a tea distribution company. This material accumulates in dust inside the equipment to pack tea into teabags. This is classified as waste in the production system and is disposed of by landfill. The collected tea dust from the confection line to pack black tea was vibratory sieve analyzed by Fritsch Analysette 3 PRO (Weimar, Germany), to separate fractions in size from 50 to 100 µm, which was further used in the preparation of composites. The moisture content of filler measured at 120 °C is 7.51%.

### 2.2. Preparation of Composites

To obtain composite materials for further pulverization to form polymer powder suitable for rotational molding technology, the materials were manufactured by mixing in a molten state. The extrusion process was performed twice using a ZAMAK EH16.2D co-rotating twin-screw extruder (Zamak Mercator, Skawina, Poland). During the first processing, the rotational speed of the screws was 100 rpm, and the maximum set temperature of the plastifying unit was 120 °C. The second extrusion process was assessed to obtain better black tea waste filler distribution in the polymeric matrix. The temperature parameters were the same as in the first extrusion, but the rotational speed of the screws was 250 rpm. The amount of the filler introduced in the bioLDPE matrix is 1, 2, 5, and 10 wt%. Materials, before all processing, were dried in a laboratory cabinet dryer Memmert ULE 500 (Schwabach, Germany) for 12 h at 60 °C. This way, prepared composites were cooled in forced airflow and pelletized. The next step was the grinding process to obtain polymer powder. For this purpose, an ultra centrifugal mill Retsch ZM 200 (Haan, Germany) equipped with a 500 µm cutting sieve was used at a rotor speed of 6000 rpm. The grinding has been improved with dry ice to cool down the rotor, sieve, and material.

**Figure 2:**
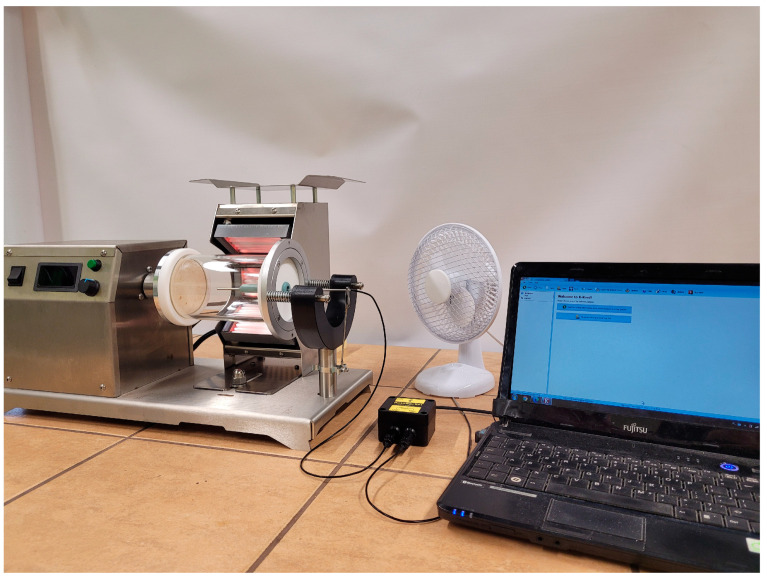
Uniaxial rotational molding station RotoRocket.

The rotational molding process was assessed using a RotoRocket desktop rotational molding machine produced by 493K (Ballyclare, UK). This is an uniaxial rotational molding machine. Temperature measurements were used as a control criterion for processing time. Moreover, the rotomolding machine is equipped with a thermocouple and can monitor and record the temperature inside the mold. The rotational molding process for all materials lasts until the temperature inside the mold reaches 200 °C then the radiant heaters are turned off and the rotational move is kept until the temperature inside the mold reaches room temperature. The rotational speed is 10 rpm. Figure 2 presents a uniaxial rotational molding station.

### 2.3. Characterization of Fillers

The optical microscope Opta-Tech MB200s connected with camera Meiji Techno HD2600T (Warsaw, Poland) was used to investigate the fillers’ and polymeric powder’s particle size distribution. The measurements were made using 200× magnification and presented results were made for at least 1000 measurements.

FTIR spectroscopic analysis was performed in a transmittance mode for black tea waste filler before and after thermal treatment to identify the chemical composition of the used filler. The mentioned thermal treatment was performed using the rotational molding machine but with only black tea waste in the mold to investigate the influence of the temperature on the filler that is similar through rotational molding. To assess this, the 4 g filler was kept in a rotational movement in RotoRocket until the temperature inside the mold reached 200 °C, then cooled. The FTIR measurement for filler before thermal treatment and after were obtained via KBr pellets using Jasco FT/IR-4600 (Tokyo, Japan) apparatus. The number of scans was 64 in a range of 4000–400 cm^−1^ with a resolution of 4 cm^−1^. The same procedure was performed to investigate the transmittance spectrum of catechin. The raw substance (+)- catechins was provided by Sigma Aldrich (Darmstadt, Germany).

The determination of L*a*b chromatic parameters according to the International Commission on Illumination (CIE) was used to evaluate the visible changes in the color of the used filler before and after thermal treatment. In this color space, L* is the color lightness (L* = 0 for black and L* = 100 for white), a* is the green (−) to red (+) axis, and b* is the blue (−) to yellow (+) axis [32]. To measure that, the portable spectrophotometer—NR145 Precision Colorimeter 3nh (Shenzhen, China) equipped with standard light illuminant D65 and aperture Φ8. Measured coordinates provided the information necessary to calculate the total color difference parameter (ΔE) [33]:

(1) $$∆E=\sqrt{{∆L}^{2}+{∆a}^{2}+{∆b}^{2}}$$

The second measured parameter is the browning index (BI) [34]. The following Equations (2) and (3) allow calculating it:

(2) $$\mathrm{BI}=\frac{100(x-0.31)}{0.17}$$

(3) $$x=\frac{\left(a^{*}+1.75L^{*}\right)}{\left(5.645L^{*}+a^{*}-0.012b^{*}\right)}$$

Antioxidant properties of the used filler were investigated using the DPPH free radical scavenging assay. The analysis was performed for fillers before and after thermal treatment. The 1 g of black tea waste was extracted in 100 mL of methanol for 30 min using the magnetic stirrer operating at 300 rpm to obtain the required extracts for testing. Then the filtered extracts were diluted to the concentration of 1 g/L. The antioxidant activity can be specified because of a stable organic nitrogen radical in 2,2-diphenyl-1-picrylhydrazyl (DPPH); its solution in alcohol has a violet color, which fades in the presence of the antioxidant. The color changes can be recorded by the UV VIS spectrophotometer, in this case a Spectrophotometer UV-VIS UviLine 9400 (Mainz, Germany) was used. The DPPH assay was performed using a 63 µM solution of DPPH in methanol [35]. In dark flasks was placed 0.15 mL of filler extract and 2.85 mL of DPPH solution was added [36]. The flasks were kept closed and stored in the dark for 30 min before measurement, which was performed at 517 nm wavelength. All measurements were performed in triplicate and expressed as a Trolox equivalent in milligrams of Trolox per gram of filler dry mass—DRSC (DPPH radical scavenging activity). The antioxidant activity can be calculated using Equation (4).

(4) $$\mathrm{AA}=\frac{A_{\mathrm{control}}-A_{\mathrm{sample}}}{A_{control}}\cdot 100\%$$

where, A_control_—absorbance of DPPH solution at 517 nm, A_sample_—absorbance of the sample at 517 nm.

### 2.4. Characterization of Composites

Color analysis was also performed for composites. The same device and a color space were used for filler. Besides the L*a*b* chromatic parameters, the browning index was calculated according to Equations (2) and (3). The total color change parameter (Equation (1)) was calculated to compare color between composites and a bioLDPE sample (ΔE_1_) and to compare color between the inner and outer surface of samples (ΔE_2_).

Fourier transform infrared spectroscopy was performed to investigate changes in the chemical structure of obtained rotomolded composites and black tea waste. The measurement was carried out using Jasco FT/IR-4600 apparatus in a wavenumber range of 4000–400 cm^−1^ at resolution 4 cm^−1^ and with 32 scans. All measurements were conducted in attenuated total reflectance mode (ATR), and their absorbance spectrum helped to calculate the carbonyl index (CI) of rotomolded parts (1):

(5) $$\mathrm{CI}=\left(\frac{A_{\left(C=O\right)}}{A_{0}}\right)$$

where: A_(C=O)_ is the absorbance of the peak of the carbonyl group (1800–1650 cm^−1^), A_0_ is the absorbance of the methylene (CH_2_)) from 1500 to 1420 cm^−1^ which was selected as an internal reference because it shows minimal affection by the thermal oxidation [37,38]. The carbonyl index is an average value of three measurements.

To identify the carbonyl band’s exact composition, deconvolution should be carried out. The second derivative method was used to locate overlapping peaks in OriginPro 9.0 software, and the deconvoluted peaks were fitted using a Gaussian profile with the accuracy of R^2^ = 0.99 also in origin software [39].

Differential scanning calorimetry (DSC) analysis was carried out to investigate changes in composite materials after incorporating black tea waste filler. Measurements were performed on a Netzsch DSC 204 F1 Phoenix (Selb, Germany) instrument under a nitrogen flow of 20 mL/min and in double cycles of heating and cooling in the temperature range of 20–210 °C at a rate of 10 °C/min. The 5 mg ± 1 mg samples were placed in aluminum pierced pans. The second heating and cooling scans were considered to further analysis. The crystallinity of obtained materials was calculated using Equation (6):

(6) $$X_{c}=\frac{∆H_{m}}{∆H_{100\%}\cdot (1-\varphi )}\cdot 100\%$$

where: ΔH_m_ is a melting enthalpy of samples [J/g], ΔH_100%_ is an enthalpy of melting PE with 100% crystalline structure, and φ is filler weight content [–] [2].

Rheological properties of the bioLDPE and composite samples were tested using a rotational rheometer Anton Paar MCR 301 (Graz, Austria) equipped with a 25 mm parallel-plate measuring system in oscillation shearing mode at a temperature of 200 °C and a gap of 0.8 mm. The preliminary strain sweep experiments were conducted before frequency sweep measurements to determine the linear viscoelastic region (LVE). Unmodified bioLDPE samples and composites containing 1–10 wt% filler were tested under constant shearing oscillatory conditions (2%; 10 rad/s) in an oxidizing atmosphere for 3600 s.

The mechanical properties of obtained rotomolded parts were examined in the tensile test. The Young modulus and tensile strength measurements test were carried out on Zwick/Roell Z010 (Ulm, Germany) according to the standard ISO 527. The cross-head speed in the range of modulus calculation was 1 mm/min and in the rest of the test 10 mm/min. The specimens used in this evaluation were 10 mm wide, 2 mm thick, and 100 mm long. Tensile strength and Young modulus are average values of at least five tests.

## 3. Results and Discussion

### 3.1. Characterization of Filler

**Figure 3:**
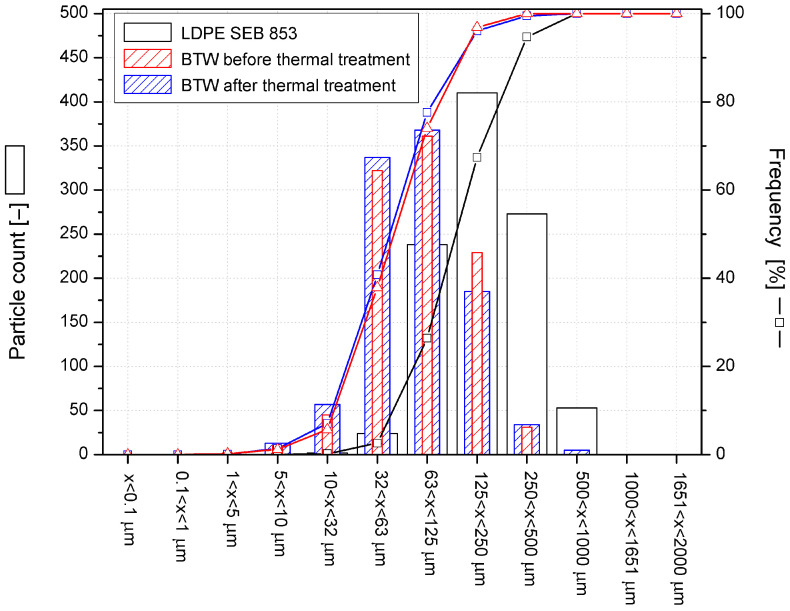
Particle size distribution of fillers and powdered polyethylene.

Figure 3 presents the results of the particle size measurement made for fillers before and after the thermal treatment process and the polyethylene after the grinding process. Based on the analysis, it can be concluded that the thermal treatment did not cause significant changes in the size of the filler particles. Therefore, the impact of the filler’s changed granulometric properties on the composite’s shaping and sintering may be omitted. In the case of polyethylene, the dominant share of particle size is in the range of 63–500 µm, which is in the range favorable from the point of view of the shaping process by rotational molding [40].

**Figure 4:**
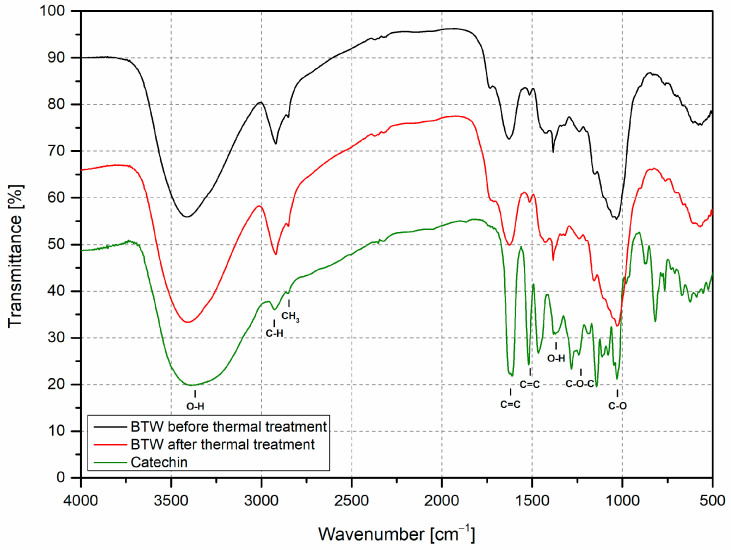
FTIR transmittance spectra of black tea waste before and after thermal treatment.

The thermal degradation of filler in temperature conditions similar to those occurring during rotational molding was analyzed using FTIR measurement. The purpose of this was to evaluate if the chemical composition of black tea waste changes and hence decreases antioxidant activity. Figure 4 presents the FTIR spectrum of black tea before and after thermal treatment. There is no visible change in the course of the transmission spectrum for both samples. All of the peaks are placed in the same position, and there is a peak at 3400 cm^−1^ linked with the O–H bond, which appears in all phenols compounds and indicates residual moisture in the sample [41]. The two following peaks correspond to the bond between C–H at 2921 cm^−1^ and CH_2_ or CH_3_ at 2852 cm^−1,^ which are present in alkanes [42]. The peaks which appear in the range 1800–1700 cm^−1^ are linked with the presence of carbonyl group (C=O); this is a characteristic bond for ester compounds that may be found in antioxidant compounds (Figure 1). In the black tea samples, this absorption band can be described as a shoulder peak, not well established and even the additional degradation in elevated temperature does not change its intensity because of a possibility of oxidation. Bands at 1625 cm^−1^ and 1513 cm^−1^ indicate the presence of aromatic skeletal since these peaks are matched with the C=C band in the aromatic ring, which exists in all antioxidant compounds present in Figure 1 and also in lignin which is a component of tea leaves [43,44]. The next peak with high intensity is 1382 cm^−1,^ responsible for band C–H which can be detected in the black tea extract spectrum [45]. The last narrow high-intensity peak at 1030 cm^−1^ is usually connected with C–O bond stretching vibrations in alcohols, esters, and carboxylic acids [46]. All other appearing peaks are small shoulder peaks at 1425 cm^−1^, 1320 cm^−1^, 1240 cm^−1,^ and 1153 cm^−1^. All of them appear in lignin FTIR spectra, respectively, for aromatic skeletal combined with C–H band in-plane deforming and stretching, CH_2_ bending stretching, C–O–C stretching, and C–H stretching [47]. The comparison of the black tea waste spectrum and catechin shows significantly a lot of the same peaks’ positions except the wide peak of H_2_O; there are similar peaks at: 1029, 1143, 1241, 1382, 1519, 1625, 2854 and 2927 cm^−1^. They are described above, and those appearing in the catechin spectrum are more narrow and have higher intensity except for the peaks related to C–H and CH_2_ stretching. All of these peaks are attributed to the bonds appearing in the catechin chemical formula.

The FTIR investigation did not provide any evidence for the thermal degradation of black tea waste that could deteriorate its antioxidant properties. However, the color change can be seen with the naked eye; the spectrophotometer was used to confirm that effect qualitatively. The average values of 11 measurement L*a*b* parameters are presented in Table 1, as well as a browning index and total color difference parameter. The browning index gives information on how brown the samples are. The black tea waste browning index is approximately 10, which is low, and since the theaflavins in black tea give an orange-red color, the browning is not at a high level [48]. The browning index increases doubles after the thermal treatment at 200 °C. The increase in the browning index denotes the darkening of black tea waste filler, which is also noticeable in the luminescence (L*) parameter value. The lower the L* value, the darker the sample. The same characteristic can be obtained after the degradation of wood samples, where the darkening is caused by the degradation of lignin [49]. In the case of black tea waste, the same phenomenon occurs since lignin is a component of black tea [43]. The total color change parameter, which exceeds 5 gives the impression of two distinct colors according to the standard PN-EN ISO/CIE 11664, and the color change between BTW before and after thermal treatment is above 25.

The last measurement performed to investigate changes in antioxidant properties of thermally treated BTW is a DPPH assay. Table 2 presents antioxidant activity values and the Trolox equivalent.

The antioxidant activity and its value calculated using Equation (4) show significant differences between them. The thermally treated one has a lesser scavenging radical ability investigated in this test resulting in a smaller equivalent of Trolox per gram of dried black tea wastes. The above-mentioned FTIR analysis did not provide any evidence of chemical composition changes in black tea waste after exposure to elevated temperatures, which was interpreted as no impact on antioxidant properties. However, the extract antioxidant activity analysis showed a deterioration of this feature three times for BTW after thermal treatment.

### 3.2. Characterization of Composites

Table 3 presents chromatic color parameters and their graphical presentation in the form of pictures of rotomolded parts surfaces. The color of rotomolded composites darkens with an increase of a filler since the L* parameter decreases. This also affects an increase in browning index only the inner surface of 10% BTW has a slightly lower BI than the 5% BTW, this can be caused by the presence of pores visible from the inner surface. The total color change for the outer and inner surface of composites compared to the neat bioLDPE exceeds 30 and ΔE > 5 gives the impression of two distinct colors but worthy of notice is the color change (ΔE_2_) between the outer and inner surface. The lowest ΔE_2_ parameter is for LDPE samples but the highest for 1% BTW, 2% BTW, and 5% BTW. The outer surface of these samples is lighter because of the presence of pores and pin holes which makes the illusion of lighter surfaces. The color analysis can also help to investigate the distribution of the filler. A total of 11 measurements were made across all outer surfaces of the samples. The standard deviation for parameters L*a*b* does not exceed 2; it is different in the case of the inner surface where the increased porosity and surface damage appears. Table 3 presents the standard deviation for color parameters and the browning index. The total color change is calculated based on an average value of L*, a*, and b* parameters.

**Figure 5:**
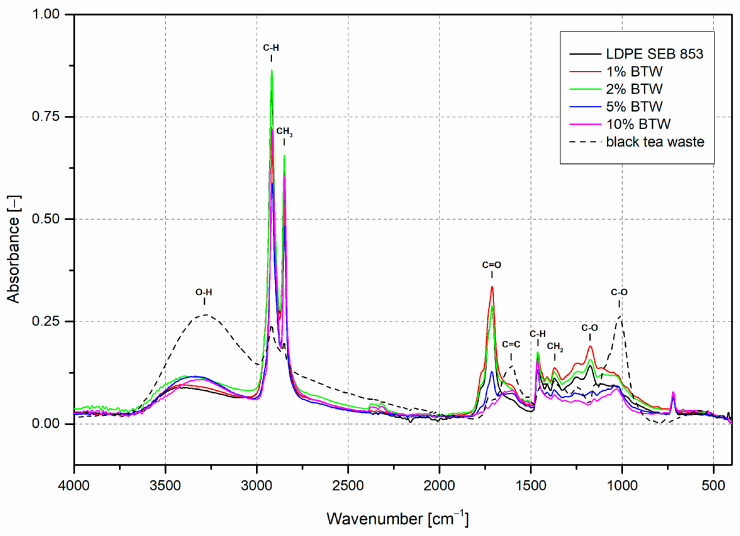
FTIR spectra of rotomolded samples and black tea waste.

**Figure 6:**
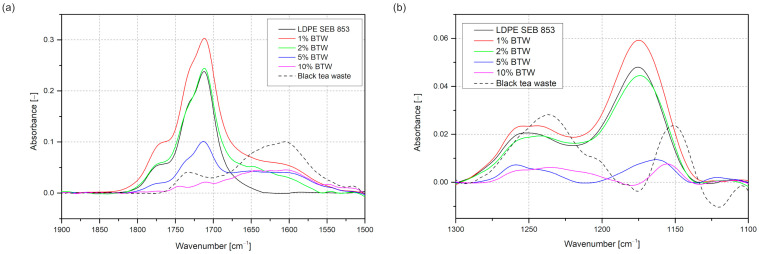
Close-up of FTIR spectra in the range: 1800–1500 cm^−1^ (**a**), 1300–1100 cm^−1^ (**b**).

FTIR spectrum of rotomolded samples is presented in Figure 5. All samples showed a wide absorption band in the range of 3100–3500 cm^−1^ with a maximum of approximately 3400 cm^−1^. This peak is associated with O–H stretching vibrations [50]. The appearance of this peak in composite materials can be connected with the addition of black tea waste, which has high absorbance at 3400 cm^−1^. The unusual behavior is presented by the bioLDPE sample; the O–H band does not appear even after long oxidation, and samples were carefully dried before processing, so this may be attributed to the usage of water-based release agents, they are commonly used in rotational molding technology [51]. Moving on to the influence of oxidation, the increase in band intensity between 1650 and 1800 cm^−1^ is observed and may be correlated with carbonyl compounds formed during the oxidation of polyethylene [52]. The close-up view of the absorption band in this range is presented in Figure 6a.

**Figure 7:**
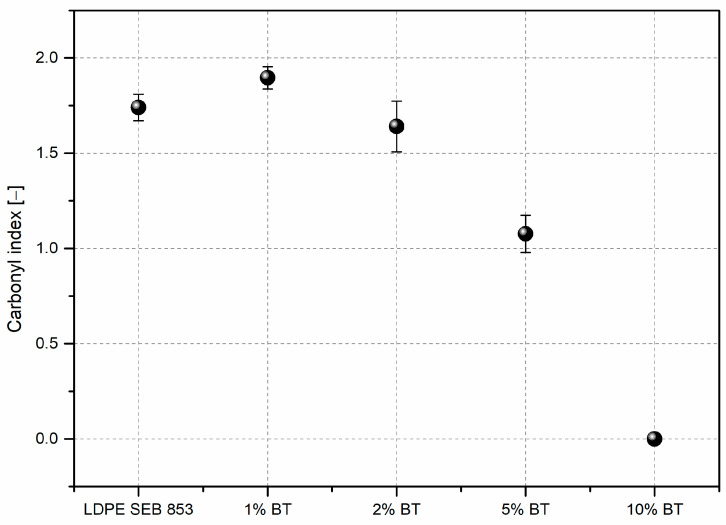
Carbonyl index values for neat LDPE and its composites.

The highest intensity of the carbonyl band has a rotomolded sample with 1 wt% of BTW, and the next is LDPE, 2% BTW, 5% BTW, and 10%, which did not have this peak. The carbonyl index (Figure 7) measurement helped to evaluate the real oxidation levels normalized by the absorption band intensity of the sample not influenced by thermal oxidation. The results obtained with this procedure agree with changes in carbonyl band intensities. Adding black tea waste in an amount of 10 wt% did not lead to the oxidation of polyethylene. The carbonyl band peak did not show up; only the increase between 1700 and 1500 cm^−1^ results from the incorporation of black tea is visible, which is associated with the C=C stretching in the aromatic ring and for C=O stretching occurring in flavonoids and catechins [42]. Thanks to the deconvolution of the carbonyl band, it is possible to specify each carbonyl compound during rotational molding. In all four samples where carbonyl peaks appear, the same compounds were distinguished: carboxylic acids (~1710 cm^−1^), esters (~1735 cm^−1^), and peresters (~1777 cm^−1^) [53]. The highest contribution has carboxylic acid and the lowest peresters.

Figure 6b presents the FTIR spectra in the wavenumber range of 1300–1100 cm^−1^, in which the peak at 1175 cm^−1^ is the C–O stretching vibration band in esters and at 1250 cm^−1^ is also the C–O stretching vibration band [54,55]. Neat bioLDPE and its composites with 1 and 2 wt% of BTW have shown high absorbance in this region. In comparison to samples where the amount of black tea waste exceeded 5 wt%, these peaks are not visible. In the ATR-FTIR spectrum of black tea waste C–O band peaks can also be distinguished. However, while the not modified LDPE also shows these peaks, they are more likely related to the oxidation of polymer than the influence of antioxidant filler.

The uniaxial rotational molding caused a high oxidation level in bioLDPE, and its composites to measure that the carbonyl index (CI) was used. Usually, the CI values did not exceed one [38,56,57] but the mentioned literature references concern the thermal and UV aging of polyethylene in the solid state. In the case of rotational molding, the sintering and further melting of polymer particles must be performed to obtain rotomolded parts. Despite the oxidizing environment during rotational molding, adding 10 wt% of black tea prevents the deterioration of polymer properties due to oxidation.

**Figure 8:**
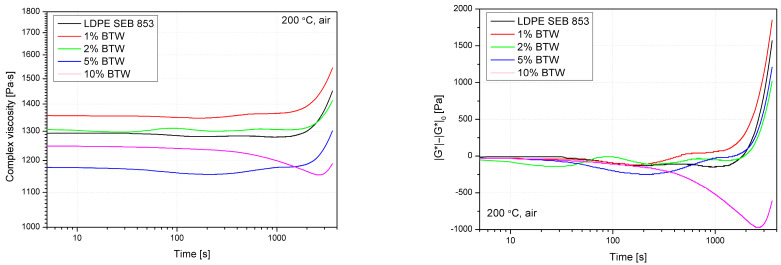
Results of stability evaluation based on viscosity measurements conducted in the oxidative atmosphere and constant shearing mode.

In order to supplement the information on the effectiveness of the BTW antioxidant effect on the polyethylene matrix, an additional rheological analysis was carried out. The measurements carried out under constant shear conditions in an oxidizing atmosphere and a temperature of 200 °C were analyzed as changes in complex viscosity and relative change of complex modulus as a function of time. Similarly presented in this study evaluation, analysis of viscosity induced by thermooxidation phenomena was described in the literature and is related to the cross-linking process of polyethylene [58,59]. Polyethylene cross-linking due to long-term temperature impact may be correlated to two mechanisms. The first one is related to the secondary alkyl reaction leading to junctions created by covalent bonds joining tertiary carbon atoms. The second mechanism mentioned in the literature is associated with the coupling of the vinylidene group with a skeleton alkyl resulting in the formation of bonds between two tertiary carbons separated by the CH_2_ group [58]. The change in the course of rheological curves obtained as a function of time (Figure 8) in elevated temperature and oxidative conditions, including the postponing of the increase in viscosity, is an additional criterion for evaluating the effectiveness of the filler’s antioxidant activity on bioLDPE. During the use of synthetic stabilizers, the preliminary decrease in viscosity is attributed to the consumption of the antioxidant, in the considered case, it will also be related to the partial degradation of the lignocellulosic filler caused by long-term exposure to elevated temperature and the release of volatiles. Despite the degradation of the structure of the lignocellulosic filler, the phytochemicals contained in the BTW retained their effectiveness, which can be observed by extending the time of the beginning of the increase in complex viscosity. Viscosity changes caused by the addition of 1% BTW results from the presence of rigid filler structures in the molten polymeric bulk, while the increasing share of the filler containing residual moisture and emitting gaseous degradation products caused an decrease in the value of the complex viscosity. The increase in the viscosity of the 5% BWT series relative to 10% BWT probably results from exceeding the threshold of rheological percolation and the hindering of the rigid filler structures [60,61,62], which opposed the melt composite structure’s porosity. In addition, the presented changes in the relative complex modulus (|G*|−|G*|_0_(t)) allow for a more apparent observation of the noticeable effect of stabilizing the polyethylene structure by the active compounds contained in the filler.

**Figure 9:**
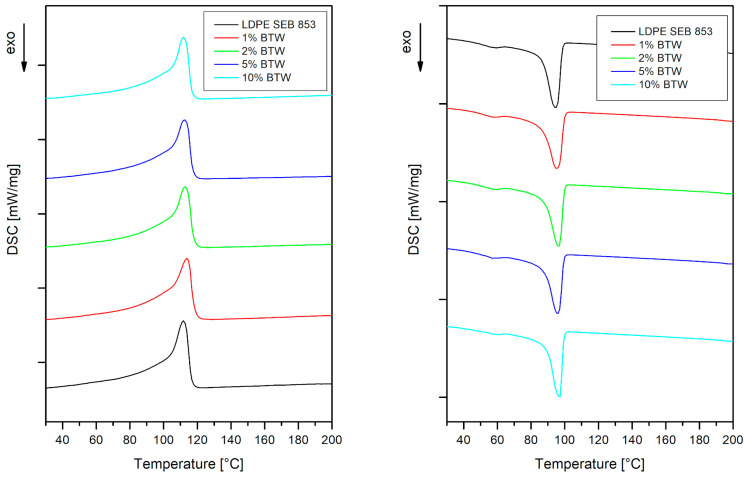
Thermograms for LDPE and composites with black tea waste.

The incorporation of black tea waste into the polymeric matrix did not provide any significant changes in the thermal properties of composites. In Figure 9 are presented endo- and exotherms of the bioLDPE sample and its composites. The melting (T_m_) and crystallization (T_c_) temperatures of neat bioLDPE did not change after adding black tea waste; all of this information is presented collectively in Table 4. This behavior suggests that the bioLDPE composites with black tea wastes can be easily processed via processing technology suitable for polyethylene without any alterations if only the processing temperatures are considered. The same characteristic presents modified LDPE with a turmeric powder, which is also an antioxidant filler and does not change the melting properties of polyethylene [63]. Since the rotomolded samples containing up to 5 wt% of filler are highly oxidized, it is expected to observe some changes in crystallinity. The amorphous phase of the material is more sensitive to thermal and UV aging, so the intensification of sample oxidation is related to an increase in crystallinity [64,65]. The sample 1% BTW has a higher carbonyl index but a lower degree of crystallinity than unmodified bioLDPE and 10% BTW which does not indicate any oxidation has a higher degree of crystallinity. It should be mentioned that according to the literature, the thermally aged samples have a higher degree of crystallinity [64].

**Figure 10:**
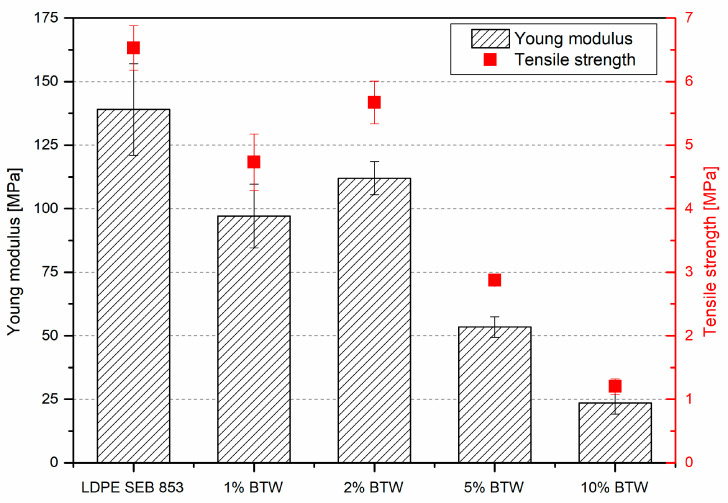
Mechanical properties of rotational molded composites samples.

Even though the addition of 10 wt% of black tea waste prevents material from oxidation but failed to provide good reinforcement of rotomolded parts. Figure 10 presents values of Young modulus and tensile strength of bioLDPE and its composites. The Young modulus decreased almost 4 times after incorporating 10 wt% of BTW compared to neat bioLDPE, and tensile strength decreased over 4.5 times. The tensile strength and Young modulus values correlate with an increase in filler content, and the trend is downward. The Young modulus of composites reinforced with powder particles should go up because of a reinforcing effect and stiffening of material. An increase in stiffness along with a decrease in tensile strength is typical behavior for thermoplastic composites reinforced with particle-shaped fillers [66]. It is different in the case of composites obtained in rotational molding technology reinforced with natural fillers. In the case of polyethylene filled with buckwheat husk in size between 50 and 200 µm, similar to the size of black tea waste, the tensile modulus does not change significantly up to 7 wt% of filler, but tensile strength decreases [2]. For natural fiber-reinforced rotomolded composites, additional surface modification should be implemented to obtain good-quality parts [8]. Low mechanical properties characterize the rotomolded composites prepared in this experiment; in comparison, the rotomolded composites with natural filler without any additional modification have tensile strength twice this value [1]. The Young modulus of rotomolded LLDPE was two times higher than the one obtained in uniaxial rotational molding [9], and the tensile strength from the material data sheet is three times higher in comparison to the experimental data. The low mechanical properties can be attributed to the insufficient quality of obtained rotomolded parts. The samples have a lot of pores that are visible to the naked eye, and with the increase of a filler, the porosity increases. According to Tian et al. [67], black tea wastes collected after tea beverage consumption subjected to the TG-FTIR measurement exhibit a mass loss between 30 and 210 °C with a maximum peak at approximately 90 °C, the same as temperature conditions for rotational molding. During thermal degradation in this temperature region, the evolved gases from black tea waste were mainly the H_2_O, CH_3_COOH, and C=C, which can form pores in material structure. The total moisture content in black tea waste can exceed 8 wt% [65]. In the rotational molding industry, it is the practice of holding mold with materials longer at elevated temperatures to allow the bubbles to migrate from the melted polymer during the densification phase of the process [68]. In this case, the increase in time could only worsen the mechanical properties. This low quality of parts can be caused by some of the evolving products from waste filler during thermal processing. To prevent this behavior, a lower processing temperature is recommended to be used.

## 4. Conclusions

Uniaxial rotational molding is a simple and quick way to test materials suitable for rotational molding on smaller scales. Adding black tea waste in the amount of 10 wt% prevents the oxidation and formation of carbonyl compounds in polyethylene. The stabilization of the polyethylene structure by the active compounds contained in the filler was also proved by the rheological analysis in a highly oxidative atmosphere. The excellent antioxidant properties of black tea are maintained by filler even after thermal treatment. The high antioxidant influence of migrating from the filler low-molecular weight active compounds on bioLDPE matrix are not only advantages. Incorporating black tea waste does not change the thermal properties of materials, as was proved by differential scanning calorimetry analysis. One disadvantage that should be addressed in future studies is the insufficient mechanical performance exhibited by the rotomolded parts. This effect is caused by a highly porous structure, which increases with the filler content. In order to balance all of these properties, samples should have been prepared at lower processing temperatures. However, considering the remarkable ability of black tea to capture free radicals and prevent the oxidation of low-density polyethylene at elevated temperatures leads instead to the search for a method to stabilize the lignocellulosic part of the filler. It has been shown in the work that the processing temperature does not adversely affect the antioxidant efficiency of the consisted in BTW active compounds in terms of the polymeric matrix stabilization.

## Figures and Tables

**Table 1:** Color parameters for black tea waste before and after thermal treatment.

	L*	a*	b*	BI	ΔE
**BTW before thermal treatment**	54.65	7.58	23.10	9.91	25.98
**BTW after thermal treatment**	29.83	8.21	15.42	19.10	

**Table 2:** Antioxidant properties of black tea waste before and after thermal treatment.

	Antioxidant Activity [%]	DRSC [mg/g Dry Mass]
BTW before thermal treatment	32.20	46.60
BTW after thermal treatment	11.50	9.16

**Table 3:** Color parameters for rotomolded composites and their pictures.

	Outer Surface						Inner Surface						ΔE_2_
	L*	a*	b*	BI	ΔE_1_	Picture	L*	a*	b*	BI	ΔE_1_	Picture	
**LDPE SEB 853**	67.08 ± 1.82	0.57 ± 0.26	3.03 ± 0.88	0.62 ± 0.25		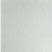	67.86 ± 1.46	0.62 ± 0.25	2.73 ± 0.83	0.63 ± 0.24		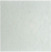	0.84
**1% BTW**	34.67 ± 1.25	7.83 ± 1.41	11.00 ± 2.20	15.80 ± 3.11	34.16	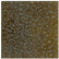	29.79 ± 3.72	10.99 ± 0.25	18.50 ± 0.99	25.43 ± 2.62	42.50	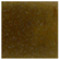	9.49
**2% BTW**	30.54 ± 0.77	6.61 ± 0.38	9.05 ± 0.34	15.12 ± 0.41	37.52	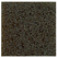	21.62 ± 1.68	9.43 ± 2.11	12.85 ± 1.95	29.06 ± 3.91	48.15	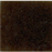	10.10
**5% BTW**	31.40 ± 1.63	6.26 ± 0.33	9.00 ± 0.41	14.02 ± 1.34	36.62	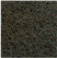	17.59 ± 0.85	6.11 ± 0.35	5.55 ± 0.43	23.72 ± 1.87	50.65	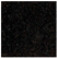	14.23
**10% BTW**	24.86 ± 1.65	3.71 ± 0.69	4.59 ± 0.83	10.62 ± 2.22	42.37	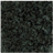	21.72 ± 1.41	4.17 ± 0.25	4.27 ± 0.38	13.49 ± 1.22	46.31	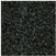	3.19

**Table 4:** Differential scanning calorimetry results for LDPE and composites.

	T_m_ [°C]	T_c_ [°C]	ΔH_m_ [J/g]	X_c_ [%]
**LDPE SEB 853**	111.8	94.4	69.55	23.74
**1% BTW**	113.8	95.2	65.31	22.52
**2% BTW**	112.7	96.3	65.05	22.65
**5% BTW**	112.4	95.8	64.16	23.05
**10% BTW**	111.8	97.0	64.08	24.30

## Data Availability

The data presented in this study are available on request from the corresponding author.
